# Bis[5-(pyridin-2-yl)pyrazine-2-carbo­nitrile-κ^2^ *N* ^4^,*N* ^5^]silver(I) perchlorate

**DOI:** 10.1107/S1600536811046708

**Published:** 2011-11-12

**Authors:** Fan Zhang, Zhi-Wei Wang, Yong-Li Yang

**Affiliations:** a Department of Chemistry, Capital Normal University, Beijing 100048, People’s Republic of China

## Abstract

In the mononuclear title complex, [Ag(C_10_H_6_N_4_)_2_]ClO_4_, the Ag^I^ ion is surrounded by two 5-(pyridin-2-yl)pyrazine-2-carbonitrile ligands, forming a considerably distorted square-planar N_4_-coordination geometry, with two short and two long Ag—N distances. Each perchlorate anion links two mononuclear coordination units through C—H⋯O(perchlorate) hydrogen bonding, forming an infinite tape structure along [110]. Inter­molecular π–π stacking inter­actions between adjacent pyridine and pyrazine rings [centroid–centroid distances of 3.777 (3) and 3.879 (2) Å] further assemble the tape motifs into a three-dimensional supra­molecular structure.

## Related literature

For coordination complexes with cyano, carboxyl­ate, pyridyl and triazole groups, see: Wang *et al.* (2009 ▶); Manriquez *et al.* (1991 ▶). For these involving 2,2′-bipyridine derivatives, see: Berghian *et al.* (2005 ▶); Mathieu *et al.* (2001 ▶). For comparable structures, see: Biju & Rajasekharan (2008 ▶); Wang *et al.* (2010 ▶). 
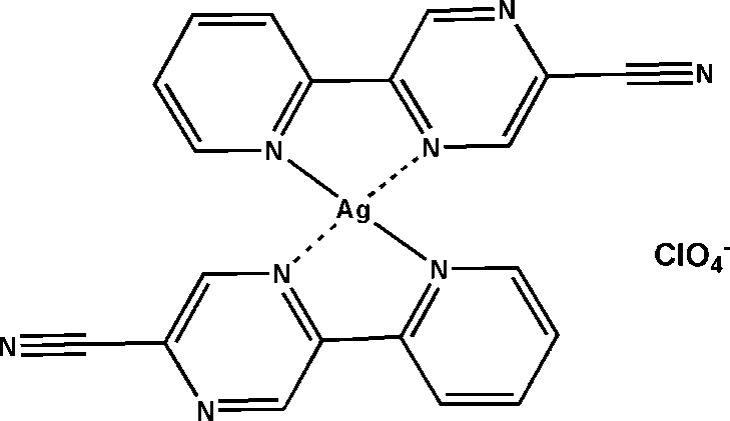


## Experimental

### 

#### Crystal data

[Ag(C_10_H_6_N_4_)_2_]ClO_4_
*M* *_r_* = 571.70
Triclinic, 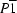

*a* = 7.8804 (10) Å
*b* = 11.3152 (14) Å
*c* = 12.3317 (14) Å
α = 104.015 (2)°
β = 92.015 (2)°
γ = 101.171 (2)°
*V* = 1042.8 (2) Å^3^
*Z* = 2
Mo *K*α radiation
μ = 1.14 mm^−1^
*T* = 293 K
0.30 × 0.20 × 0.12 mm

#### Data collection

Bruker APEXII CCD area-detector diffractometer
Absorption correction: multi-scan (*SADABS*; Bruker, 2007 ▶) *T*_min_ = 0.577, *T*_max_ = 0.755
7304 measured reflections
5075 independent reflections
3882 reflections with *I* > 2σ(*I*)
*R*_int_ = 0.021

#### Refinement

*R*[*F* ^2^ > 2σ(*F* ^2^)] = 0.034
*wR*(*F* ^2^) = 0.092
*S* = 1.03
5075 reflections
307 parameters
10 restraints
H-atom parameters constrained
Δρ_max_ = 0.54 e Å^−3^
Δρ_min_ = −0.31 e Å^−3^

### 

Data collection: *APEX2* (Bruker, 2007 ▶); cell refinement: *APEX2* and *SAINT* (Bruker, 2007 ▶); data reduction: *SAINT*; program(s) used to solve structure: *SHELXS97* (Sheldrick, 2008 ▶); program(s) used to refine structure: *SHELXL97* (Sheldrick, 2008 ▶); molecular graphics: *SHELXTL* (Sheldrick, 2008 ▶); software used to prepare material for publication: *SHELXTL* and *PLATON* (Spek, 2009 ▶).

## Supplementary Material

Crystal structure: contains datablock(s) I, global. DOI: 10.1107/S1600536811046708/ez2265sup1.cif

Structure factors: contains datablock(s) I. DOI: 10.1107/S1600536811046708/ez2265Isup2.hkl

Additional supplementary materials:  crystallographic information; 3D view; checkCIF report

## Figures and Tables

**Table 1:** Selected bond lengths (Å)

Ag1—N1	2.184 (2)
Ag1—N5	2.193 (2)
Ag1—N6	2.683 (2)
Ag1—N2	2.739 (2)

**Table 2:** Hydrogen-bond geometry (Å, °)

*D*—H⋯*A*	*D*—H	H⋯*A*	*D*⋯*A*	*D*—H⋯*A*
C15—H15*A*⋯O2^i^	0.93	2.71	3.203 (2)	114
C14—H14*A*⋯O2^i^	0.93	2.54	3.103 (2)	119
C5—H5*A*⋯O4^ii^	0.93	2.45	3.193 (3)	137

Symmetry codes: (i) 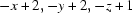; (ii) 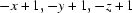.

**Fig. 1.**
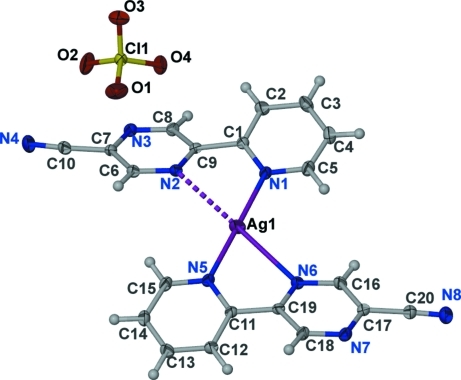
The atom-numbering scheme of the title [Ag(C10H6N4)2]ClO4. Displacement ellipsoids are drawn at the 30% probability level.

**Fig. 2.**
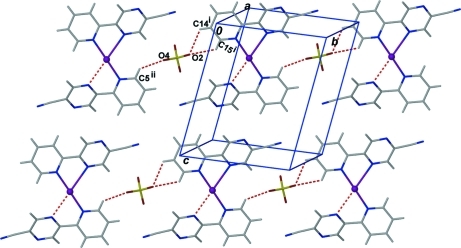
The perchlorate linkages between the [Ag(C10H6N4)2]+ moieties arranged along the [110] direction. The red-dashed lines indicate the C—H···O(perchlorate) Hydrogen-bonding interactions, while the purple balls represent the AgI ions. Symmetry codes: i –x + 2, –y + 2, –z + 1; ii –x + 1, –y + 1, –z + 1.

**Fig. 3.**
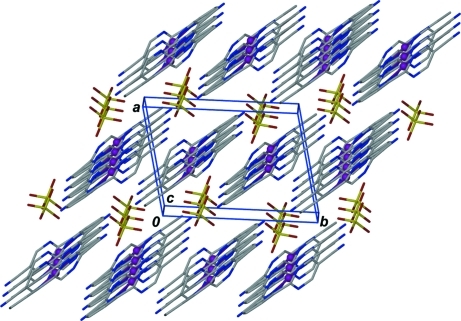
View down the c axis of the three-dimensional supramolecular structure of the title complex. All non-covalent interactions are omitted for clarity.

## supplementary crystallographic information

### Comment

Cyano, carboxylate, pyridyl and triazole groups have been widely employed as organic linkers to bond with metal ions to construct subtile metal organic frameworks (MOFs) (Wang *et al.* 2009; Manriquez *et al.* 1991). Many 2,2'-bipyridine derivatives together with their various metal complexes have also been synthesized and well characterized (Berghian *et al.* 2005; Mathieu *et al.* 2001).

Herein, we present the structure of a new complex [Ag(C_10_H_6_N_4_)_2_]ClO_4_ derived from 5-(2-pyridyl)pyrazine-2-carbonitrile, a similar ligand to the 2,2'-bipyridine featuring a 2-cyanopyrazinyl group bonding to the 2-pyridyl carbon atom (Scheme 1). As shown in Fig. 1, the two ligands around the central Ag^I^ ion are in an anti-relationship and almost in the same plane, thus the Ag^I^ ion is surrounded by two 2-pyridyl N atoms and two 2-pyrazinyl N atoms. The Ag1—N1(pyridyl) and Ag1—N5(pyridyl) bonds are 2.184 (2) and 2.193 (2) Å, respectively. Meanwhile, the longer Ag1—N6(pyrazinyl) and Ag1—N2(pyrazinyl) distances are 2.684 (2) Å and 2.739 (3) Å, respectively. The Ag—N bond lengths are similar to those (2.196 (2)–2.685 (2) Å) in the isomorphous mononuclear structure of [Ag(C_10_H_6_N_4_)_2_]BF_4_ reported by us recently (Wang *et al.*, 2010). Also, the longer Ag—N(pyrazinyl) distance is comparable to that in [Ag(dafone)_2_]NO_3_.H_2_O (dafone = 4,5-diazafluoren-9-one) (Biju & Rajasekharan, 2008). If the weak Ag···N contact is included, a planar N4-square coordination geometry is formed. The perchlorate anions function as linkages to link neighboring [Ag(C_10_H_6_N_4_)_2_]^+^ moieties arranged along the [110] direction into an infinite tape structure through C—H···O interactions (Table 1, Fig. 2). The tapes are stacked along the [110] direction and interconnect via π–π interactions. The Cg1(pyridyl)···Cg1^i^(pyridyl) and Cg2(pyridyl)···Cg2^ii^(pyridyl) distances are 3.777 (3) and 3.879 (2) Å, respectively, while that of Cg3(pyrazinyl)···Cg3^iii^(pyrazinyl) is 3.626 (2) Å (symmetry codes: I –x + 1, –y + 1, –z + 1; ii –x + 1, –y + 2, –z + 2; iii –x + 1, –y + 2, –z + 1. Cg1, Cg2, Cg3 represent the N1-C1-C2-C3-C4-C5, N5-C11-C12-C13-C14-C15 and N2-C6-C7-N3-C8-C9 rings, respectively). A three-dimensional supramolecular framework is formed (Fig. 3).

### Experimental

The ligand 5-(2-pyridyl)-2-cyanopyrazine was obtained commercially. To a clear solution of 3 ml methanol containing the ligand (18.2 mg, 0.1 mmol), AgClO_4_ (22 mg, 0.1mmol) was added with stirring at room temperature. 1 ml acetonitrile was subsequently added dropwise to make the solution clear. After filtration the clear solution was kept in air for one week at room temperature to yield colorless rod-like crystals (19.0 mg, 66% yeild).

### Refinement

All the H atoms were discernible in the difference electron density maps. Nevertheless, the hydrogen atoms were placed into idealized positions and allowed to ride on the carrier atoms, with C—H = 0.93 Å for aryl hydrogens.*U*_iso_(H) = 1.2*U*_eq_(C)_aryl_.

### Crystal data

**Table:**

[Ag(C_10_H_6_N_4_)_2_]ClO_4_	*Z* = 2
*M**_r_* = 571.70	*F*(000) = 568
Triclinic, *P*1	*D*_x_ = 1.821 Mg m^−^^3^
*a* = 7.8804 (10) Å	Mo *K*α radiation, λ = 0.71073 Å
*b* = 11.3152 (14) Å	Cell parameters from 233 reflections
*c* = 12.3317 (14) Å	θ = 1.7–28.2°
α = 104.015 (2)°	µ = 1.14 mm^−^^1^
β = 92.015 (2)°	*T* = 293 K
γ = 101.171 (2)°	Rod, colorless
*V* = 1042.8 (2) Å^3^	0.30 × 0.20 × 0.12 mm

### Data collection

**Table:**

Bruker APEXII CCD area-detector diffractometer	5075 independent reflections
Radiation source: fine-focus sealed tube	3882 reflections with *I* > 2σ(*I*)
graphite	*R*_int_ = 0.021
ω scans	θ_max_ = 28.3°, θ_min_ = 1.7°
Absorption correction: multi-scan (*SADABS*; Bruker, 2007)	*h* = −10→10
*T*_min_ = 0.577, *T*_max_ = 0.755	*k* = −15→10
7304 measured reflections	*l* = −16→15

### Refinement

**Table:**

Refinement on *F*^2^	Primary atom site location: structure-invariant direct methods
Least-squares matrix: full	Secondary atom site location: difference Fourier map
*R*[*F*^2^ > 2σ(*F*^2^)] = 0.034	Hydrogen site location: inferred from neighbouring sites
*wR*(*F*^2^) = 0.092	H-atom parameters constrained
*S* = 1.03	*w* = 1/[σ^2^(*F*_o_^2^) + (0.0409*P*)^2^ + 0.4461*P*] where *P* = (*F*_o_^2^ + 2*F*_c_^2^)/3
5075 reflections	(Δ/σ)_max_ = 0.001
307 parameters	Δρ_max_ = 0.54 e Å^−^^3^
10 restraints	Δρ_min_ = −0.31 e Å^−^^3^

### Special details

**Table:**

Geometry. All esds (except the esd in the dihedral angle between two l.s. planes) are estimated using the full covariance matrix. The cell esds are taken into account individually in the estimation of esds in distances, angles and torsion angles; correlations between esds in cell parameters are only used when they are defined by crystal symmetry. An approximate (isotropic) treatment of cell esds is used for estimating esds involving l.s. planes.
Refinement. Refinement of F^2^ against ALL reflections. The weighted R-factor wR and goodness of fit S are based on F^2^, conventional R-factors R are based on F, with F set to zero for negative F^2^. The threshold expression of F^2^ > 2sigma(F^2^) is used only for calculating R-factors(gt) etc. and is not relevant to the choice of reflections for refinement. R-factors based on F^2^ are statistically about twice as large as those based on F, and R- factors based on ALL data will be even larger.

### Fractional atomic coordinates and isotropic or equivalent isotropic displacement parameters (Å^2^)

**Table:**

	*x*	*y*	*z*	*U*_iso_*/*U*_eq_	
Ag1	0.50428 (3)	0.75075 (2)	0.705977 (16)	0.05505 (10)	
C1	0.3777 (3)	0.6399 (2)	0.45026 (19)	0.0332 (5)	
C2	0.2969 (4)	0.5507 (3)	0.3561 (2)	0.0460 (6)	
H2A	0.3090	0.5652	0.2854	0.055*	
C3	0.1978 (4)	0.4399 (3)	0.3670 (3)	0.0519 (7)	
H3A	0.1420	0.3794	0.3042	0.062*	
C4	0.1834 (4)	0.4209 (3)	0.4728 (3)	0.0519 (7)	
H4A	0.1166	0.3478	0.4832	0.062*	
C5	0.2700 (4)	0.5124 (3)	0.5623 (2)	0.0490 (7)	
H5A	0.2616	0.4985	0.6334	0.059*	
N1	0.3662 (3)	0.6209 (2)	0.55367 (17)	0.0380 (5)	
C6	0.7085 (3)	0.9257 (2)	0.5006 (2)	0.0392 (6)	
H6A	0.8006	0.9708	0.5527	0.047*	
C7	0.6760 (3)	0.9659 (2)	0.4053 (2)	0.0369 (5)	
N3	0.5409 (3)	0.9082 (2)	0.32982 (17)	0.0391 (5)	
C9	0.4813 (3)	0.7606 (2)	0.43988 (19)	0.0323 (5)	
N2	0.6093 (3)	0.8232 (2)	0.51831 (16)	0.0371 (5)	
C8	0.4446 (3)	0.8066 (2)	0.3479 (2)	0.0367 (5)	
H8A	0.3490	0.7641	0.2977	0.044*	
C11	0.6248 (3)	0.8669 (2)	0.96211 (18)	0.0329 (5)	
C12	0.7024 (4)	0.9591 (3)	1.0561 (2)	0.0411 (6)	
H12A	0.6862	0.9473	1.1274	0.049*	
C13	0.8034 (4)	1.0681 (3)	1.0438 (2)	0.0451 (6)	
H13A	0.8560	1.1306	1.1063	0.054*	
C14	0.8250 (4)	1.0828 (3)	0.9377 (2)	0.0500 (7)	
H14A	0.8927	1.1552	0.9266	0.060*	
C15	0.7445 (4)	0.9883 (3)	0.8481 (2)	0.0519 (7)	
H15A	0.7598	0.9990	0.7764	0.062*	
N5	0.6457 (3)	0.8821 (2)	0.85757 (17)	0.0410 (5)	
C16	0.2892 (4)	0.5835 (2)	0.9077 (2)	0.0435 (6)	
H16A	0.2074	0.5338	0.8504	0.052*	
C17	0.2979 (3)	0.5534 (2)	1.0099 (2)	0.0372 (5)	
C18	0.5251 (3)	0.7139 (3)	1.0743 (2)	0.0400 (6)	
H18A	0.6126	0.7593	1.1296	0.048*	
C19	0.5123 (3)	0.7507 (2)	0.97452 (18)	0.0322 (5)	
N6	0.3962 (3)	0.6827 (2)	0.89007 (17)	0.0408 (5)	
N7	0.4176 (3)	0.6168 (2)	1.09313 (17)	0.0418 (5)	
C10	0.7861 (4)	1.0716 (3)	0.3789 (2)	0.0464 (6)	
N4	0.8670 (4)	1.1518 (3)	0.3512 (2)	0.0716 (8)	
C20	0.1815 (4)	0.4486 (3)	1.0339 (2)	0.0443 (6)	
N8	0.0976 (4)	0.3679 (3)	1.0585 (2)	0.0603 (7)	
Cl1	0.94869 (8)	0.75080 (6)	0.28153 (5)	0.03921 (15)	
O3	1.0442 (3)	0.6770 (2)	0.20672 (19)	0.0635 (6)	
O4	0.7780 (3)	0.6817 (2)	0.28566 (19)	0.0590 (5)	
O1	1.0367 (3)	0.7906 (3)	0.39031 (19)	0.0854 (9)	
O2	0.9306 (4)	0.8567 (2)	0.2423 (3)	0.0870 (9)	

### Atomic displacement parameters (Å^2^)

**Table:**

	*U*^11^	*U*^22^	*U*^33^	*U*^12^	*U*^13^	*U*^23^
Ag1	0.07966 (19)	0.05148 (15)	0.02647 (11)	0.00307 (12)	−0.00799 (10)	0.00575 (9)
C1	0.0370 (12)	0.0342 (12)	0.0289 (11)	0.0064 (10)	0.0016 (9)	0.0099 (10)
C2	0.0593 (17)	0.0446 (15)	0.0304 (12)	0.0041 (13)	−0.0068 (11)	0.0091 (11)
C3	0.0600 (18)	0.0374 (15)	0.0489 (16)	−0.0027 (13)	−0.0132 (13)	0.0056 (12)
C4	0.0540 (17)	0.0401 (15)	0.0598 (18)	−0.0018 (13)	−0.0004 (14)	0.0193 (14)
C5	0.0591 (17)	0.0481 (16)	0.0400 (14)	0.0006 (14)	0.0055 (13)	0.0193 (13)
N1	0.0451 (12)	0.0390 (12)	0.0289 (10)	0.0022 (9)	0.0030 (9)	0.0117 (9)
C6	0.0460 (14)	0.0367 (13)	0.0292 (12)	−0.0005 (11)	−0.0004 (10)	0.0050 (10)
C7	0.0488 (14)	0.0298 (12)	0.0313 (12)	0.0067 (11)	0.0096 (10)	0.0066 (10)
N3	0.0446 (12)	0.0408 (12)	0.0325 (10)	0.0070 (10)	0.0033 (9)	0.0121 (9)
C9	0.0372 (12)	0.0334 (12)	0.0262 (11)	0.0077 (10)	0.0068 (9)	0.0065 (9)
N2	0.0467 (12)	0.0356 (11)	0.0253 (9)	0.0030 (9)	0.0006 (8)	0.0059 (8)
C8	0.0366 (13)	0.0405 (14)	0.0332 (12)	0.0054 (11)	0.0011 (10)	0.0120 (10)
C11	0.0368 (12)	0.0357 (12)	0.0254 (11)	0.0056 (10)	0.0014 (9)	0.0082 (9)
C12	0.0528 (15)	0.0419 (14)	0.0263 (11)	0.0048 (12)	−0.0005 (10)	0.0088 (10)
C13	0.0519 (16)	0.0398 (14)	0.0368 (13)	0.0011 (12)	−0.0043 (11)	0.0046 (11)
C14	0.0541 (16)	0.0428 (15)	0.0477 (16)	−0.0068 (13)	0.0048 (13)	0.0148 (13)
C15	0.0649 (18)	0.0529 (17)	0.0338 (14)	−0.0052 (14)	0.0080 (13)	0.0169 (13)
N5	0.0500 (12)	0.0434 (12)	0.0256 (10)	−0.0018 (10)	0.0025 (9)	0.0105 (9)
C16	0.0543 (16)	0.0384 (14)	0.0308 (12)	−0.0020 (12)	−0.0032 (11)	0.0057 (11)
C17	0.0410 (13)	0.0353 (13)	0.0347 (12)	0.0074 (11)	0.0080 (10)	0.0078 (10)
C18	0.0428 (14)	0.0448 (15)	0.0299 (12)	−0.0008 (11)	−0.0004 (10)	0.0133 (11)
C19	0.0373 (12)	0.0346 (12)	0.0239 (10)	0.0075 (10)	0.0030 (9)	0.0059 (9)
N6	0.0534 (13)	0.0370 (12)	0.0271 (10)	−0.0004 (10)	−0.0008 (9)	0.0070 (9)
N7	0.0473 (12)	0.0455 (13)	0.0320 (11)	0.0027 (10)	0.0029 (9)	0.0140 (10)
C10	0.0627 (17)	0.0367 (14)	0.0330 (13)	−0.0002 (13)	0.0008 (12)	0.0051 (11)
N4	0.104 (2)	0.0470 (16)	0.0509 (16)	−0.0174 (16)	0.0052 (15)	0.0140 (13)
C20	0.0489 (15)	0.0419 (15)	0.0389 (14)	0.0029 (12)	0.0045 (12)	0.0092 (12)
N8	0.0701 (17)	0.0492 (15)	0.0560 (16)	−0.0049 (13)	0.0054 (13)	0.0166 (13)
Cl1	0.0416 (3)	0.0371 (3)	0.0345 (3)	0.0008 (3)	0.0034 (2)	0.0066 (2)
O3	0.0616 (13)	0.0711 (15)	0.0531 (13)	0.0254 (12)	0.0021 (10)	−0.0022 (11)
O4	0.0473 (11)	0.0565 (13)	0.0687 (14)	−0.0064 (10)	0.0009 (10)	0.0215 (11)
O1	0.0658 (15)	0.121 (2)	0.0418 (12)	−0.0060 (15)	−0.0094 (11)	−0.0089 (14)
O2	0.0997 (19)	0.0640 (16)	0.126 (2)	0.0313 (14)	0.0595 (18)	0.0591 (17)

### Geometric parameters (Å, °)

**Table:**

Ag1—N1	2.184 (2)	C11—C12	1.387 (3)
Ag1—N5	2.193 (2)	C11—C19	1.481 (3)
Ag1—N6	2.683 (2)	C12—C13	1.378 (4)
Ag1—N2	2.739 (2)	C12—H12A	0.9300
C1—N1	1.347 (3)	C13—C14	1.371 (4)
C1—C2	1.379 (4)	C13—H13A	0.9300
C1—C9	1.486 (3)	C14—C15	1.372 (4)
C2—C3	1.382 (4)	C14—H14A	0.9300
C2—H2A	0.9300	C15—N5	1.333 (3)
C3—C4	1.378 (4)	C15—H15A	0.9300
C3—H3A	0.9300	C16—N6	1.335 (3)
C4—C5	1.369 (4)	C16—C17	1.386 (3)
C4—H4A	0.9300	C16—H16A	0.9300
C5—N1	1.342 (3)	C17—N7	1.331 (3)
C5—H5A	0.9300	C17—C20	1.450 (4)
C6—N2	1.337 (3)	C18—N7	1.325 (3)
C6—C7	1.392 (3)	C18—C19	1.397 (3)
C6—H6A	0.9300	C18—H18A	0.9300
C7—N3	1.339 (3)	C19—N6	1.336 (3)
C7—C10	1.447 (4)	C10—N4	1.135 (4)
N3—C8	1.321 (3)	C20—N8	1.131 (4)
C9—N2	1.333 (3)	Cl1—O1	1.416 (2)
C9—C8	1.401 (3)	Cl1—O2	1.426 (2)
C8—H8A	0.9300	Cl1—O3	1.426 (2)
C11—N5	1.353 (3)	Cl1—O4	1.428 (2)
			
N1—Ag1—N5	179.23 (7)	C13—C12—H12A	120.0
N1—C1—C2	121.7 (2)	C11—C12—H12A	120.0
N1—C1—C9	117.9 (2)	C14—C13—C12	118.7 (3)
C2—C1—C9	120.4 (2)	C14—C13—H13A	120.7
C1—C2—C3	119.9 (2)	C12—C13—H13A	120.7
C1—C2—H2A	120.1	C13—C14—C15	118.6 (3)
C3—C2—H2A	120.1	C13—C14—H14A	120.7
C4—C3—C2	118.7 (3)	C15—C14—H14A	120.7
C4—C3—H3A	120.7	N5—C15—C14	124.0 (2)
C2—C3—H3A	120.7	N5—C15—H15A	118.0
C5—C4—C3	118.3 (3)	C14—C15—H15A	118.0
C5—C4—H4A	120.9	C15—N5—C11	117.7 (2)
C3—C4—H4A	120.9	C15—N5—Ag1	118.68 (17)
N1—C5—C4	124.0 (3)	C11—N5—Ag1	123.31 (17)
N1—C5—H5A	118.0	N6—C16—C17	121.1 (2)
C4—C5—H5A	118.0	N6—C16—H16A	119.5
C5—N1—C1	117.5 (2)	C17—C16—H16A	119.5
C5—N1—Ag1	118.25 (17)	N7—C17—C16	122.4 (2)
C1—N1—Ag1	124.21 (17)	N7—C17—C20	114.5 (2)
N2—C6—C7	121.0 (2)	C16—C17—C20	123.0 (2)
N2—C6—H6A	119.5	N7—C18—C19	122.6 (2)
C7—C6—H6A	119.5	N7—C18—H18A	118.7
N3—C7—C6	122.3 (2)	C19—C18—H18A	118.7
N3—C7—C10	114.7 (2)	N6—C19—C18	120.4 (2)
C6—C7—C10	123.0 (2)	N6—C19—C11	119.3 (2)
C8—N3—C7	116.1 (2)	C18—C19—C11	120.3 (2)
N2—C9—C8	120.9 (2)	C16—N6—C19	117.3 (2)
N2—C9—C1	119.2 (2)	C18—N7—C17	116.1 (2)
C8—C9—C1	119.9 (2)	N4—C10—C7	175.5 (3)
C9—N2—C6	117.1 (2)	N8—C20—C17	175.7 (3)
N3—C8—C9	122.4 (2)	O1—Cl1—O2	109.5 (2)
N3—C8—H8A	118.8	O1—Cl1—O3	109.86 (16)
C9—C8—H8A	118.8	O2—Cl1—O3	109.58 (15)
N5—C11—C12	121.1 (2)	O1—Cl1—O4	109.76 (14)
N5—C11—C19	118.5 (2)	O2—Cl1—O4	107.38 (15)
C12—C11—C19	120.4 (2)	O3—Cl1—O4	110.68 (14)
C13—C12—C11	120.0 (2)		
			
N1—C1—C2—C3	1.5 (4)	C11—C12—C13—C14	0.0 (4)
C9—C1—C2—C3	−178.6 (3)	C12—C13—C14—C15	0.2 (5)
C1—C2—C3—C4	−0.5 (5)	C13—C14—C15—N5	0.0 (5)
C2—C3—C4—C5	−0.8 (5)	C14—C15—N5—C11	−0.4 (5)
C3—C4—C5—N1	1.2 (5)	C14—C15—N5—Ag1	173.3 (2)
C4—C5—N1—C1	−0.2 (4)	C12—C11—N5—C15	0.6 (4)
C4—C5—N1—Ag1	−177.0 (2)	C19—C11—N5—C15	178.6 (2)
C2—C1—N1—C5	−1.2 (4)	C12—C11—N5—Ag1	−172.76 (19)
C9—C1—N1—C5	178.9 (2)	C19—C11—N5—Ag1	5.3 (3)
C2—C1—N1—Ag1	175.5 (2)	N1—Ag1—N5—C15	28 (6)
C9—C1—N1—Ag1	−4.4 (3)	N1—Ag1—N5—C11	−159 (6)
N5—Ag1—N1—C5	151 (6)	N6—C16—C17—N7	−3.6 (4)
N5—Ag1—N1—C1	−26 (6)	N6—C16—C17—C20	178.4 (3)
N2—C6—C7—N3	3.0 (4)	N7—C18—C19—N6	−4.7 (4)
N2—C6—C7—C10	−176.0 (2)	N7—C18—C19—C11	174.1 (2)
C6—C7—N3—C8	−3.3 (4)	N5—C11—C19—N6	−19.7 (3)
C10—C7—N3—C8	175.8 (2)	C12—C11—C19—N6	158.4 (2)
N1—C1—C9—N2	26.0 (3)	N5—C11—C19—C18	161.5 (2)
C2—C1—C9—N2	−153.9 (2)	C12—C11—C19—C18	−20.5 (4)
N1—C1—C9—C8	−154.5 (2)	C17—C16—N6—C19	0.5 (4)
C2—C1—C9—C8	25.6 (4)	C18—C19—N6—C16	3.4 (4)
C8—C9—N2—C6	−5.1 (3)	C11—C19—N6—C16	−175.4 (2)
C1—C9—N2—C6	174.4 (2)	C19—C18—N7—C17	1.6 (4)
C7—C6—N2—C9	1.4 (4)	C16—C17—N7—C18	2.4 (4)
C7—N3—C8—C9	−0.5 (4)	C20—C17—N7—C18	−179.4 (2)
N2—C9—C8—N3	4.9 (4)	N3—C7—C10—N4	−14 (4)
C1—C9—C8—N3	−174.6 (2)	C6—C7—C10—N4	165 (4)
N5—C11—C12—C13	−0.4 (4)	N7—C17—C20—N8	−11 (4)
C19—C11—C12—C13	−178.4 (2)	C16—C17—C20—N8	167 (4)

### Hydrogen-bond geometry (Å, °)

**Table:**

*D*—H···*A*	*D*—H	H···*A*	*D*···*A*	*D*—H···*A*
C15—H15A···O2^i^	0.93	2.71	3.203 (2)	114
C14—H14A···O2^i^	0.93	2.54	3.103 (2)	119
C5—H5A···O4^ii^	0.93	2.45	3.193 (3)	137

Symmetry codes: (i) −*x*+2, −*y*+2, −*z*+1; (ii) −*x*+1, −*y*+1, −*z*+1.
